# Prevalence of and Risk Factors for Childhood Asthma, Rhinitis, and Eczema in Hong Kong: Proposal for a Cross-Sectional Survey

**DOI:** 10.2196/resprot.7252

**Published:** 2017-06-07

**Authors:** So Lun Lee, Yu Lung Lau, Hing Sang Wong, Linwei Tian

**Affiliations:** ^1^ Department of Paediatrics & Adolescent Medicine Li Ka Shing Faculty of Medicine The University of Hong Kong Hong Kong China (Hong Kong); ^2^ Department of Paediatrics The Duchess of Kent Children's Hospital at Sandy Bay Hong Kong China (Hong Kong); ^3^ The University of Hong Kong - Shenzhen Hospital Shenzhen China; ^4^ School of Public Health Li Ka Shing Faculty of Medicine The University of Hong Kong Hing Kong China (Hong Kong)

**Keywords:** child health, ISAAC, air pollution, asthma, eczema, rhinitis

## Abstract

**Background:**

Previous studies have shown that particulate matter is a major problem in indoor air quality in Hong Kong schools, but little has been done to assess its relationship with health indicators in the children attending those schools. Our study aims to address this research gap by collecting aerosol data in schools to examine the link between different air pollutants with childhood respiratory health. It is important to explore whether or not the prevalence of asthma, allergic rhinoconjunctivitis, and eczema are increasing in local children.

**Objective:**

Our aim is to (1) examine the prevalence of asthma, allergic rhinitis, and eczema in school children aged 6-7 years in Hong Kong between 2001 and 2017, and (2) measure air quality at primary schools and explore its relationship with health outcomes measured by the International Study of Asthma and Allergies in Childhood (ISAAC) survey.

**Methods:**

This is a cross-sectional study consisting of an ISAAC questionnaire and aerosol data collection. We have recruited over 2000 parents of primary school students aged 6-7 years old for the questionnaire, and so far 19 schools have completed aerosol data collection.

**Results:**

The study is expected to be completed this year.

**Conclusions:**

We predict that our study will show a significant change in the prevalence of asthma, allergic rhinitis, and eczema in school children aged 6-7 years old in recent years. In addition, we expect to show a significant association between air quality at school and health outcomes measured by the ISAAC survey.

## Introduction

The prevalence of asthma and allergic diseases has increased substantially in past decades, imposing a significant disease burden on patients, their families, and society [1-5]. Yet recent studies indicate wide global variation in the prevalence of asthma, allergic rhinoconjunctivitis (AR), and eczema, with a growing trend in developing countries and a plateau or even a decreasing trend in developed countries [6]. It has been speculated that environmental and lifestyle factors may continue to induce asthma symptoms in susceptible individuals until the saturation level in prevalence, determined by the genetic composition of the population, is achieved [7]. Local surveys are essential to assess disease burden in terms of prevalence, economic impact, and effects on quality of life in order to prioritize resource allocation.

The International Study of Asthma and Allergies in Childhood (ISAAC) is the largest worldwide collaborative research project, involving more than 100 countries and nearly 2 million children. It was designed to compare the prevalence of these disorders between populations in different countries, thereby forming the basis for studies investigating the role of possible modifiable environmental factors that may lead to a reduction in the burden of these diseases [8]. Our department (Department of Paediatrics & Adolescent Medicine, Queen Mary Hospital/University of Hong Kong) participated in Phase 1 of ISAAC in 1995, which showed that the prevalence of allergic disorders in school children aged 6-7 years old in Hong Kong was comparable to that in Singapore and Great Britain [9]. We then participated in Phase 3 of ISAAC in 2001, which showed there had been a significant increase in prevalence of severe asthma symptoms, life-time rhinitis, current rhinitis, and life-time eczema and a seeming plateau of life-time asthma, life-time wheeze, and current wheeze [10]. Similar findings were evident in children aged 13-14 years old [11]. We also attempted to explore the change in environmental factors over the period, which was adopted from the environmental module of ISAAC; this may account for the change in prevalence of serve asthma symptoms, life-time rhinitis, current rhinitis, and life-time eczema. Risk factors identified included history of wheezing in parents, male sex, frequent upper respiratory tract infection in early life, and month of birth in locally born girls. However, the increase in prevalence of rhinitis, eczema, and severe asthma symptoms could not be entirely explained by the change of prevalence of the aforementioned risk factors, suggesting that some unidentified factors in Hong Kong might have also contributed significantly to the observed change.

In a subsequent cohort study in 2004, we found that viral infection, a well-known trigger, could account for only a third of asthma exacerbations in 114 children with stable asthma over a 1-year period [12]. Similarly, another local case-control study could only identify significant respiratory pathogens in half of the children admitted for asthma exacerbations [13]. We believed that environmental air pollution was a factor for asthma exacerbation. We confirmed our speculation through another study, showing the significant association between air pollution and asthma admission, an indicator for severe asthma among children in Hong Kong [14]. This study was also the first study in the world that showed the relationship between asthma admission with fine particles PM_2.5_, now recognized as an important air pollutant in different parts of the world. Based on this finding, a reduction of ambient levels of air pollutants including NO_2_, PM_10_, and ozone by an average of 50% of those levels recorded during the 6-year study period could have prevented 570 hospital admissions for asthma in children each year. A recent local study also showed that coarse PM (PMc; 2.5-10 microgram aerodynamic diameter) was associated with emergency hospital admissions for respiratory diseases in Hong Kong independent of PM_2.5_ and gaseous pollutants [15]. In Hong Kong, road traffic, together with the “Canyon effect” due to multistory buildings, is a major source of air pollution especially at roadside in Hong Kong. Exposure to traffic emissions is generally unavoidable in children living in urban areas. Epidemiologic studies have reported associations between residential proximity to busy roads and adverse respiratory health outcomes in children, including respiratory symptoms, asthma exacerbations, and decrements in lung function [16]. A local study confirmed that daily particulate air pollution levels (PM_10_) in children’s living locations derived from surface extinction coefficients (SEC), as measured by satellite and measurements from air pollutant monitoring stations at ground level, were associated with increased odds of having respiratory symptoms (cough or sputum) [17]. In Hong Kong, while exposure to air pollutants in residential areas poses a major concern for child health, it is noteworthy that 20% of 650 primary schools in Hong Kong are situated close to a main road (as defined by the Transport Department), at a mean distance of 20.5 meters [16].

In industrialized countries, children spend more time (up to 80%) indoors, mostly in schools, than in other places except home. Studies in the United States, South Korea, and Europe have shown poor indoor air quality in many schools and its association with short-term and long-term health problems, including both respiratory and non-respiratory issues [18-20]. A local study in the late 1990s showed that particulate matter was a major problem of indoor air quality in five schools being monitored, but there was no attempt to assess its relationship with health indicators in the children attending those schools [21].

It has been more than a decade since Phrase 3 of ISAAC was conducted in Hong Kong. It is important to explore whether or not the prevalence of asthma, AR, and eczema continue to increase in local children. As ISAAC is a school-based type of survey, it will provide a good opportunity to study the air quality in schools and its relationship to health outcomes measured by the survey. We believed that the result may provide additional information for planning of child health care in Hong Kong.

### Aims and Hypotheses

Our aim is to (1) examine the prevalence of asthma, AR, and eczema in 4000 school children aged 6-7 years in Hong Kong between 2001 and 2017, and (2) measure air quality at primary schools and explore its relationship with health outcomes measured by the ISAAC survey.

We hypothesize that (1) there has been a significant change in the prevalence of asthma, allergic rhinitis, and eczema in school children aged 6-7 years old from the last survey in 2001, and (2) there is a significant association between air quality at school and health outcomes measured by the ISAAC survey.

## Methods

### ISAAC Questionnaire

ISAAC is a world-recognized study using a questionnaire to assess respiratory disease. Our research team has already used this questionnaire for several studies in Hong Kong [9,10]. The survey results were comparable with other countries and have been validated in previous studies.

The questionnaire survey will be the same as the study conducted in 1995 and 2001, that is, the same sampling frame, age group, sample size, questionnaire, and translation.

#### Selection of Subjects

Our focus is on school children aged 6-7 years old in Hong Kong. Every co-educational primary school in Hong Kong will be divided into 18 groups according to district. Then, within each district, each eligible school will be allocated a number, and two schools will be selected from each district using a table of random numbers. Once a school has been chosen, two school grades will be selected based on having the greatest proportion of 6-7 year olds. All classes in the two grades will be included. We recognize that there will be some children outside the specified age range in each class chosen. These children will be included in the data collection excluded from analysis for the international comparison. If a selected school refuses to participate, it will be replaced by another chosen at random. No eligible children will be excluded from the sample. Based on the previous study, 25 schools will provide an adequate number of children to undertake the study. We plan to recruit 36 schools as the class size may have shrunk over the past few years. We estimate that we will recruit at least 4000 participants.

#### Sample Size and Power Considerations

The prevalence of symptoms of asthma, AR, and itchy rash were 9.4%, 37.4%, and 4.2% respectively in a study population of 4448 in our previous study. A sample size of 4000 will be able to detect annual increase (decrease) of 0.5% (-0.4%) in prevalence of symptoms of asthma and other allergic diseases for at least 5 years after our last study with a power of 90% at the 5% level of significance.

#### Study Design

This study is cross-sectional. Three 1-page core questionnaires were developed by the ISAAC to ensure comparable information on the basic epidemiology of asthma, AR, and eczema and can be compared temporally and among countries. The Chinese translation of the questionnaire has been standardized. Additional questions are allowed at the end of the core questionnaire to identify potential environmental risk factors. Four new questions will be added to the set of environmental questions used in 2001 to ask for participants’ residential location including the floor of the residential building and the amount of time participants spend at school and at home.

The research was approved by the Institutional Review Board of the University of Hong Kong/Hospital Authority Hong Kong West Cluster.

#### Timeline

This study will be undertaken in 2015 to 2017. We will conduct a pilot test to test new questions at the pediatric outpatient department of Queen Mary Hospital in Hong Kong. Parents of children attending the clinic will be invited to participate.

#### Participation

A participation rate of at least 90% will be sought. It is a concern that absent children may be away from school because of asthma or allergies. Therefore, strenuous efforts need to be made to contact these children and offer them the opportunity to participate in the study. Extra questionnaires will be left in each school for the absent students to take home once they return to school. We will remind the school every month for unreturned questionnaires until we receive all answered questionnaires or until the end of the data collection period. In any case where consent has been refused, demographic data (age, sex, ethnic group) from the school will be sought.

#### Data Handling and Quality Control

The completed questionnaires will not be changed under any circumstances. Data will be entered in the computer exactly as recorded on the completed questionnaire. A coding manual is available from ISAAC so the core questions will be coded in a standard manner. Any changes to data should be done so for an explicit reason and documented and a copy of original data kept. The questionnaire must not be altered for consistency between the stem and following questions. If some questions are left blank on a particular questionnaire, it will be at the discretion of the ISAAC international data center as to whether that questionnaire is excluded for international comparison. We shall be responsible for coding our own data and data entry as well as analysis. Data are entered two times, and the two versions of the dataset will be compared and any differences checked against the original questionnaire. Any inconsistency can be resolved at that time based on the original questionnaire. A copy of data required for international comparisons will be sent to the ISAAC international data center for analysis along with data from other centers.

**Procedures**

A randomly selected list of schools will be generated. The school secretary of each school will be contacted by telephone and a personalized letter sent. The letter will include a sample of the questionnaire and the information letter (with the consent form attached) to parents/guardians. All contact with the schools, dates of phone calls, dates that letters are sent, and names of all contact people, especially the secretary will be recorded. The school principal will be called 1 week after the letter has been sent to discuss the procedure that would be undertaken for approval to be given.

When permission has been granted, an appointment will be made for the research assistant to visit the school. The school will be instructed to make available a class list with all the children’s names, ages, dates of birth, and ethnic origins, if possible.

As part of the study, we will prepare an appropriate number of reminder stickers to put on the bottom of the information letter (eg, “Please return by Friday” or another day), giving them about a week to complete and return the questionnaire to school. Stickers will be prepared for the children as a small token when the questionnaire is returned. On the day of distribution of the questionnaire, we will give a small introduction of the project to members of the school staff. The number of classes and number of students in each class in Primary 1 and 2 must be obtained, as well as the center and school number (center and school numbers can be entered later as long as Step IV is completed). The name of school must stamped onto each questionnaire (stamp borrowed from school), and the letter to parents enclosed inside the questionnaire (sticker with date of return put on the bottom of the information letter).

Before distribution, we will perform a final check to ensure that the questionnaire has been numbered, and school name stamped, and then prepare bundles for each classroom held with 2 rubber bands (one horizontal and one vertical) and the class room number written on the top of the questionnaire. A sufficient numbers of stickers will accompany classroom questionnaires and a note attached to each bundle of questionnaires, thanking the teacher. The decision when to return to collect the questionnaires will be discussed with the secretary. The timing will be approximately 1 week.

When we return to the school, all surveys will be sorted based on number sequence and marked off on the class list. For the numbers not marked off, another questionnaire will be re-issued using the same process as above, but the survey number will be 2 instead of 1. A stamped self-addressed envelope will be given for the second issues. For surveys returned uncompleted or blank, we will re-issue the survey but put a survey number 2 above the survey number 1. It will be noted on the class lists the surveys not returned and those returned blank, as well as a record of the re-issue date. A few spare stamped self-addressed envelopes will be left with the school secretary, with a request that any questionnaires returned late to the school be mailed back to the researcher/fieldworker. A letter of thanks will be given to the school, and we will advise them that they will be notified of the results when available.

The surveys collected are put into a locked filing cabinet. Any returned by post are checked against the class list and put into the appropriate class by number sequence. At a less pressured time, the surveys can be checked against the class lists for correct date of birth, age, and numbering. Any corrections to demographic data must be recorded. The questionnaire must not be altered under any circumstances.

### School Air Quality Monitor

School visits will be conducted during class time in the academic year. Three sets of air quality monitors will be used for each school: two inside 2 different classrooms and another in an open area within the school. Air pollutants will be monitored at each school for 2 consecutive weeks (5 school days per week). One of the classrooms in each grade level (Primary One and Two) in the school will be selected randomly for the placement of the indoor air quality monitors. Concentrations of particulate matter (PM_10_ and PM_2.5_) will be measured using filter-based samples collected by a PM_2.5_ monitor (TSI AM510) charged with a pump. Concentrations of nitrogen dioxide (NO_2_), ozone (O_3_), and sulphur dioxide (SO_2_) will be measured by passive diffusion samplers using nitrogen dioxide monitor (Z-1400XP), ozone monitor ZDL-1200, and sulfur dioxide monitor (Z-1300), respectively. Temperature and humidity data will also be collected. Safe and childproof sampling sites will be ensured and will comply with the rules as prescribed by the International Organization for Standardization [22,23]. Indoor samples will be collected at a height of about 1-1.5 m above the floor, which is the breathing zone inside the classroom. The selected place will not be allowed to be closer than 1 m to a wall, door, or air-conditioner. Furthermore, the indoor sampling site will be selected as far away as possible to the blackboard, taking into account a minimum distance of 3 m. Outdoor samples will be collected on an open playground if available or near the main school gate. This site will be documented. The monitors will be kept at heights of 1.5-2 m above the ground, at a minimum distance of 1 m from the closest building. Class teachers will be invited to fill out a questionnaire about the school building characteristics (eg, floor of the classroom, classroom adjacent to playground or street) and the indoor characteristics of the classroom during the study period (eg, number of students attending class, ventilation habits, such as whether windows are kept open or closed and the average number of times windows are opened to ventilate the classroom, type of blackboard for teaching, the presence of air conditioning). We will go to the school to download the information from the monitor every week for 2 consecutive weeks.

### Satellite Data

Satellite data in this study will be taken from the Moderate Resolution Imaging Spectroradiometer (MODIS) sensors aboard two NASA satellites, Aqua and Terra. Data retrievals are done based on a collection of 3 MODIS level 1B data. An algorithm is used to derive aerosol optical depth (AOD) at a resolution of 1 km x 1 km from the MODIS data based on MODIS 2.1-µm reflectance with 99% cloud-free exclusion criteria on pixels [24]. SEC, which represent surface aerosol distribution, are then derived from the AOD data by taking into account the atmospheric moisture level, the boundary layer height as derived from micropulse lidar measurement [25], and surface visibility reports. Ongoing data retrieval and derivation of AOD and SEC have been conducted by Hong Kong University of Science and Technology. The processed data have been continuously extracted by the University of Hong Kong through a Wellcome Trust project in the School of Public Health and are freely available online.

### Estimation of Ambient Air Quality at School Year-Round

Our air quality monitors will measure air pollutant levels at each school for 2 consecutive weeks. Association of our outdoor air quality data with SEC and AOD data from NASA satellites and air quality data from the nearest monitoring stations of the Environmental Protection Department (EPD) Hong Kong will be determined. Using this association, we will estimate school outdoor air quality for a whole year. The satellite data from EPD are freely available online.

### Estimation of Ambient Air Quality at Residential Locations

We will ask the participants to provide their residential address in the questionnaire. We will then geocode their residential locations into geographical coordinates using Google Maps. SEC and AOD derived from NASA satellite data will be used to estimate the residential ambient particulate air quality based on the residential locations. This information will be used to adjust for the variation of exposure at home. The building floor number will also be asked to estimate the approximate height from ground level to further adjust for the proximity from road traffic emissions on the ground level.

### Data Processing and Analysis

To assess time trend and environmental factors that account for change, any missing answers on the questionnaire will be merged with negative answers in the analysis, according to ISAAC instructions. Statistical analysis will include percentages, odds ratios, 95% confidence intervals, and chi-square tests for comparison of prevalence in 2001 and this study. All data will be calculated using SAS PC. A significance level of *P*<.05 will be used for all analyses.

We will then assess the relationship between school air quality with asthma and AR. For each pollutant, a 10-day mean concentration (mg/m^3^) in each classroom will be computed and a three-class variable of exposure (high, medium, or low) will be defined with respect to the tertiles of the concentration distribution in the classrooms, independent of the district. To investigate the impact of air quality in classrooms, health outcomes will include those measured by ISAAC questionnaires. Potential confounders will be assessed through the same environmental questionnaires in 2001 together with some new questions as previously described. These will include age, sex, place of birth, perinatal factors, ethnicity, number of siblings, socioeconomic status as indicated by highest level of maternal education attained, family income, family size, area of living quarter, the type of accommodation, hospital admission for respiratory tract infection in the first year of life and preceding year, passive smoking, parental or maternal history of asthma and allergic diseases, keeping of pets, and type of fuel used for cooking. The newly added questions will help assess average time at home and at school, road traffic on residential street, and presence of air conditioning and indoor air purifiers at children’s homes. Exposure of air pollution at home will also be adjusted from the estimation of ambient air quality at residential locations. Between-school and within-school variability (school variance and classroom variance respectively) of the measured indoor pollutants will be estimated using linear models for longitudinal data (SAS MIXED procedure). Comparisons between groups for population characteristics (ie, within or without a certain health outcome) will be made using the Student *t* test for continuous variables and the Pearson chi-square test or exact Fisher test for the categorical variables. To investigate the relationship between each health outcome and each air pollutant, a marginal model will be used [20] taking into account the potential confounders and air quality around residence by satellite data. This model will calculate the adjusted odds ratio for each health outcome at different levels of pollutant exposure. The marginal model parameters will be estimated using the generalized estimating equation approach (SAS GENMOD) with independent working correlation structure using the individual school number as stratum. SAS v.9.1 will be used for statistical analyses. Bonferroni adjustment will be used to reduce type I errors and tackle the multitasking issue. *P*<.05 will indicate statistical significance.

## Discussion

### Principal Considerations

Our study aims to examine the prevalence of asthma, AR, and eczema in 4000 school children aged 6-7 years in Hong Kong between 2001 and 2017, as well as to measure air quality at primary schools and explores its relationship with health outcomes measured by the ISAAC survey. The result is useful for understanding the prevalence of childhood asthma and allergies and identifies possible risk factors. It is useful for health care planners in estimating the needs of related services and designing possible prevention measures. Association between school air quality and respiratory health if present will help to inform related departments on planning urban land use.

### Limitations

With only 2 years scheduled for data collection and a limited budget, the research team could collect aerosol information in each school for only 2 weeks at three different sites. In Hong Kong, most of the schools have installed air conditioners and they are used for most of the year. Therefore, the seasonal difference would be mitigated. To address this critical issue, data collected from the nearest air monitoring station and satellite data would also be drawn to correlate with the data collected from schools to predict the annual indoor air quality and the variation during different seasons.

This is a cross-sectional study; as such, it is limited in its ability to predict future outcomes or detail past changes. Nevertheless, we will compare the result from this study with the 2001 ISAAC study in Hong Kong. As our study focuses on a questionnaire, it is also susceptible to recall bias. Missing data will likely occur, either because of parental refusal or questions being left blank. We will reduce this as much as possible by re-issuing questionnaires and reminding the schools to return missing questionnaires.

### Conclusion

We are now approaching the final stage of the study with only a few schools remaining for data collection. We are continually processing the data, and it is anticipated that the result will be available at the end of 2017 or early 2018.

## Appendix

Multimedia Appendix 1 Reviewers' and Grant Review Board comments on the protocol.

## Abbreviations

AOD: aerosol optical depth
AR: allergic rhinoconjunctivitis
EPD: Environmental Protection Department
ISAAC: International Study of Asthma and Allergies in Childhood
MODIS: Moderate Resolution Imaging Spectroradiometer
NO2: nitrogen dioxide
O3: ozone
PM: PMc; 2.5-10 microgram aerodynamic diameter
SEC: surface extinction coefficient
SO2: sulphur dioxide
