# Supplementary material for: Prevalence of and Risk Factors for Childhood Asthma, Rhinitis, and Eczema in Hong Kong: Proposal for a Cross-Sectional Survey
Source: JMIR Res Protoc. 2017 Jun 7;6(6):e106. doi: 10.2196/resprot.7252 (PMC5480011; doi:10.2196/resprot.7252)
Supplement: Multimedia Appendix 1 [file resprot_v6i6e106_app1.pdf]

Research Council
Grant Review Board

GRB ASSESSMENT REPORT – GRANT APPLICATION

**Project No. :**	**12134021**
**Project Title :**	**Prevalence and risk factors of childhood asthma, allergic rhinitis and eczema in – three surveys from 1995 to 2014**
**Principal Applicant (PA) :**	**Dr LEE So-lun**
**Rating :**	**4. Recommended for support**

GRB comments

1. Pilot the new questions before implementing them in the study.
2. It is appropriate to use multi-level modelling but the team should use "school" rather than "district" as stratum. Multiple schools per district would be chosen and school level factors are most likely to induce correlation in outcomes between students.
3. Consider adjusting the analysis by the socioeconomic status of the family and not just maternal education.
4. Consider the risk of Type I errors as there would be a large number of outcomes to be assessed.
5. Provide evidence of regulatory approval (including survey research ethics).
6. An audit fee of $5,000 should be included under "Other Expenses".
7. A governance system to adequately monitor the disbursement of cash or cash vouchers to ensure accountability and traceability is required.
8. Budget items Ozone monitor (ZDL-1200) ($59,520) and Sulphur Dioxide monitor (ZDL-1300) ($49,560) should be included under "Equipment" instead of "Other Expenses". Please submit a revised budget as per the template under the point-by-point response.
9. The group led by Dr Lee has several proposals recommended for support in the current open call:
   02133316 – [Functional analysis and evaluation of ultrastructure of respiratory cilia in healthy Chinese children in Hong Kong]
   12134021 – [Prevalence and risk factors of childhood asthma, allergic rhinitis and eczema in – three surveys from 1995 to 2014]
   Provide assurance that each project can be completed according to the proposed timeline and resources. Justify the expenses for these projects on consumables and manpower in more detail and clarify if any savings can be obtained from conducting these studies in parallel if they are subsequently approved

Grading for applications	Meaning
4<br>Recommended for support	Nil or very minor issues to address only
3<br>Recommended for support subject to clarifications/ amendments	Minor revision and clarification required for a successful delivery
2<br>Not recommended for support at present	Major revision required for significant improvement
1<br>Not worthy of support	Minimal impact on research / flaw in methodology/ incomplete application/ out of scope of the fund

Research Fund Secretariat
Research Office, Food and Health Bureau
9/F, Rumsey Street Multi-storey Carpark Building, 2 Rumsey Street, Sheung Wan, Hong Kong
Tel: (852) 3150 8987 Fax: (852) 2102 2444 E-mail:rfs@fhb.gov.hk

Research Council
Grant Review Board

Reviewers' comments

- The Reviewers' comments are attached for information only. No action is required.

Action required by PA

- Respond to the Grant Review Board's comments above point-by-point.
- Submit a revised "Proposed Research Project", i.e. Section 13 of Application Form, as pdf files (in "Track Changes" mode and a clean copy).
- A cover letter detailing the response to the above items. A checklist for the revised application is attached for your reference.
- Return all the materials required by **6 October 2014**. The regulatory/ethics approvals shall be submitted no later than **14 November 2014**.

Grading for applications	Meaning
4<br>Recommended for support	Nil or very minor issues to address only
3<br>Recommended for support subject to clarifications/ amendments	Minor revision and clarification required for a successful delivery
2<br>Not recommended for support at present	Major revision required for significant improvement
1<br>Not worthy of support	Minimal impact on research / flaw in methodology/ incomplete application/ out of scope of the fund

REFEREE'S ASSESSMENT FORM

Reference No.: 12134021

Project Title: Prevalence and risk factors of childhood asthma, allergic rhinitis and eczema in – three surveys from 1995 to 2014

PART A: REFEREE'S DETAILED REMARKS ON THE INDIVIDUAL SECTIONS OF THE GRANT APPLICATION

This part, except Question 9, will be forwarded to the applicant for refining and improving the proposal. The identity of the reviewer will not be released to the applicant. Please provide as much detail as possible to assist the applicant and the Grant Review Board.
Please comment on the proposed research in the following aspects

1. **Originality and Impact** What is the importance of the proposed research in terms of its originality and potential impact in the area under study? Is the proposed work original and important and will it influence practice?

The study is a regular periodic survey of asthma and upper respiratory tract conditions in children 6-7 years. While the idea is not original, it is of public health impact. Periodic public health surveillance of disease prevalence is still needed for asthma and respiratory conditions. It will affect the public health resource allocation and prevention efforts.

2. **Research Questions, Aims and Hypotheses** How specific, clearly expressed and realistic are the research questions, aims and hypotheses?

The research questions were clearly sated, and the aims are clearer compared with the previous submission. The hypotheses were stated, however, the potential changes between the current time period versus 2001 wave of survey and underlying reasons were not detailed for a repeat of the survey. Certainly about 13-14 passed, and an update of the information is needed.

3. **Subjects and Study Methodology** (i) Is the proposed design and methodology appropriate for the study? (ii) Are sample sizes clear, justified, adequate and realistic? (iii) Are any preliminary data available? (iv) How feasible is the proposed timeframe?

The design was appropriate, and the sample size is clearly stated. According to the experience of the investigators, the sample size was achievable. The preliminary data from previous surveys were loosely mentioned, but no doubt the study can be conducted in the time frame.<br><br>The use of different estimates of air pollution is to commend. However, sampling of air pollutants in different schools could be at different time/season, so comparability between schools is difficult. Satellite data has long-term changes and may be a better indicator of mean exposure.

4. **Outcomes and Data Analysis** (i) Are the primary and secondary outcomes clearly defined? (ii) Have potential problems been anticipated and addressed? (iii) Is the statistical/analytical design appropriate and clearly explained?

The outcomes were outlined and the questionnaire was attached. The potential problems about absenteeism of sick children were addressed. Clustering of subjects was mentioned and statistical analysis was planned for this clustering effect. Repeated measurements will provide contrast with prior surveys.

5. **Research Capability** Comment on (i) the research team's expertise and track record (incl. principal investigator / project team members / collaborators) and (ii) the existing facilities of the Institution where the research will be conducted.

The researchers are capable to conduct this study, and the facilities are good enough.

6. **Budget** Is the request for research personnel, consumables, equipment and overall budget justified and reasonable?

Reasonable

7. **Ethical and Safety Considerations** Is the proposed research ethically sound? Outline any safety or ethical issues that from the proposed research and comment on whether these have been adequately addressed in the proposal. Has ethical approval been sought?

No concerns.

8. **Overall Comments and Conclusion** It is always helpful for applicants to receive constructive feedback from reviewers. What are the specific strengths and weaknesses of this proposal? Please include a brief overall appraisal of the proposal focusing on any areas for improvement and the basis for your comments, e.g. awareness of other work in the field.

Strengths:

1. Large repeated survey of respiratory condition prevalence in children
2. Prior work in this area conveys assurance of the study team

Weaknesses:

1. The effort to identify the role of air pollution on the prevalence is hindered by cross-sectional nature of the study, without long-term follow-up of same children

Reviewer 2

12134021

REFEREE'S ASSESSMENT FORM

Reference No.: 12134021

Project Title: Prevalence and risk factors of childhood asthma, allergic rhinitis and eczema in – three surveys from 1995 to 2014

PART A: REFEREE'S DETAILED REMARKS ON THE INDIVIDUAL SECTIONS OF THE GRANT APPLICATION

This part, except Question 9, will be forwarded to the applicant for refining and improving the proposal. The identity of the reviewer will not be released to the applicant. Please provide as much detail as possible to assist the applicant and the Grant Review Board.
Please comment on the proposed research in the following aspects

1. **Originality and Impact** What is the importance of the proposed research in terms of its originality and potential impact in the area under study? Is the proposed work original and important and will it influence practice?

The proposed study addresses a number of important public health concerns including temporal trends in the prevalence of asthma, allowing for international comparisons through the ISAAC survey, and the association between air quality and respiratory health in children. Assessing the prevalence of asthma could help determine the level of healthcare resource required for the local population with respect to asthma, eczema and allergic rhinitis. Assessing the association between air quality and respiratory health may provide valuable information for determining the potential impact of school air quality on child respiratory health and potentially could influence the location of schools and regulation of air quality in and around schools.

2. **Research Questions, Aims and Hypotheses** How specific, clearly expressed and realistic are the research questions, aims and hypotheses?

The research questions are clear with respect to assessing the prevalence of asthma. Concerning the association between school air quality and respiratory health the research question is clear in terms of the included population, exposures and study design but not so clear on the main outcomes. The investigators should ideally decide a priori their primary and secondary outcomes.

3. **Subjects and Study Methodology** (i) Is the proposed design and methodology appropriate for the study? (ii) Are sample sizes clear, justified, adequate and realistic? (iii) Are any preliminary data available? (iv) How feasible is the proposed timeframe?

i) The proposed design and methods are appropriate for the study. However it should be noted that this is a cross-sectional survey and as such cannot be used to definitively infer causality. Furthermore the lack of prospective design makes it harder to make any inferences about the association between air quality and the prevalence of asthma.

ii) Sample sizes are clear, justified, adequate and realistic.

iii) This study builds on previous ISAAC surveys conducted in 1995 and 2001, using similar methods.

iv) The proposed timeframe seems feasible.

4. **Outcomes and Data Analysis** (i) Are the primary and secondary outcomes clearly defined? (ii) Have potential problems been anticipated and addressed? (iii) Is the statistical/analytical design appropriate and clearly explained?

i) The primary outcome is clearly defined for assessing the prevalence of asthma, eczema and allergic rhinitis. However it is less well defined for the assessment of the association between air quality and respiratory health. There are a large number of potential outcomes that could be used and the investigators should ideally select their primary and secondary outcomes a priori to avoid type I errors. However this should not stop the investigators conducting other exploratory analyses.

ii) Potential problems have been anticipated such as questionnaire non response and sensible procedures to address these have been reported.

iii) There are potential problems with multiple statistical testing that may arise due to the large number of outcomes. This could be partly addressed by specifying a priori the primary and secondary outcomes in relation to the assessment of the association between school air quality and respiratory health. The investigators should explain how they will account for potential type I errors that may arise from multiple testing.
I'm unclear what the investigators mean when they refer to use of a 'marginal model' but I am assuming this is a logistic regression model with a random effects (i.e. multilevel) component to account for potential clustering by school or district.

5. **Research Capability** Comment on (i) the research team's expertise and track record (incl. principal investigator / project team members / collaborators) and (ii) the existing facilities of the Institution where the research will be conducted.

i) The research team appear to have the necessary expertise and were involved in a previous ISAAC survey which involved similar methods to the proposed study.

ii) The existing facilities at the institution where the research will be conducted appear to be satisfactory.

6. **Budget** Is the request for research personnel, consumables, equipment and overall budget justified and reasonable?

The request for the overall budget and resources seems reasonable and justified.

7. **Ethical and Safety Considerations** Is the proposed research ethically sound? Outline any safety or ethical issues that from the proposed research and comment on whether these have been adequately addressed in the proposal. Has ethical approval been sought?

The proposed research appears to have been reviewed by an ethics committee with a favourable outcome. The research methods are non-invasive and procedures have been described that would maintain participant confidentiality. I have only one concern in this area and this relates to one particular sentence in the parent/guardian questionnaire: "We will also want to see if there is any impact of the school environment on their respiratory health problem." This may be interpreted as suggesting that the participant has a respiratory problem, which could be alarming to parents, particularly since the majority of children in the study will not have respiratory disease. I think this sentence should be revised so that this is clarified (however it may be that this is already the case in the Cantonese version).

8. **Overall Comments and Conclusion** It is always helpful for applicants to receive constructive feedback from reviewers. What are the specific strengths and weaknesses of this proposal? Please include a brief overall appraisal of the proposal focusing on any areas for improvement and the basis for your comments, e.g. awareness of other work in the field.

Strengths:

This study addresses an important public health problem, is supported by a team that are experienced in the subject matter, and has the potential to influence policy on air quality and location of schools. Data on important confounders such as air quality at residential areas is being collected and a number of important pollutants are being measured in a standardised way across the enrolled schools. The study aims to acquire a large sample size, which should provide plenty of scope for secondary analyses. The data will be captured using a standardised questionnaire used by other ISAAC surveys which will allow for international comparisons.

Weaknesses:

The cross-sectional design has inherent limitations with respect to inferences on causality. Also the case ascertainment of asthma, eczema and allergic rhinitis are based on parental self-report, which will not account for misdiagnosis and under-diagnosis. These issues cannot be overcome with the current study design and the investigators have acknowledged the first issue but should also acknowledge the second issue (outcome ascertainment) in the subsequent reports. There are potential issues with multiple statistical testing which may be partly overcome by specifying primary and secondary outcomes a priori. The investigators should also consider how they will account/correct for multiple testing. There is also no mention of whether the additional questions added to the ISAAC questionnaire have been piloted- this should be performed before the main survey is conducted.

REFEREE'S ASSESSMENT FORM

Reference No.: 12134021

Project Title: Prevalence and risk factors of childhood asthma, allergic rhinitis and eczema in – three surveys from 1995 to 2014

PART A: REFEREE'S DETAILED REMARKS ON THE INDIVIDUAL SECTIONS OF THE GRANT APPLICATION

This part, except Question 9, will be forwarded to the applicant for refining and improving the proposal. The identity of the reviewer will not be released to the applicant. Please provide as much detail as possible to assist the applicant and the Grant Review Board.
Please comment on the proposed research in the following aspects

1. **Originality and Impact** What is the importance of the proposed research in terms of its originality and potential impact in the area under study? Is the proposed work original and important and will it influence practice?

> Studying time trends in asthma prevalence is important because asthma is becoming a large worldwide problem. Few locations have three time trend points, so this is original work. You found an increase of asthma prevalence in the last study and an association between air pollution and asthma admission in another study. Did those results have any impact? If so, it could be commented on reinforce the importance of study again.

2. **Research Questions, Aims and Hypotheses** How specific, clearly expressed and realistic are the research questions, aims and hypotheses?

> They are really clear, with the exception of air quality at home. In the abstract, the aim is expressed as "Association between air quality at school and at home with respiratory health of the participants will be assessed".
> Maybe you could add to aim "2. To measure air quality at primary school and to explore its relationship with the health outcomes measured by ISAAC survey, adjusted by air quality estimation at home".
> Moreover, in the abstract you say "School air quality will be monitored for 5 days...", but in the text (section "school air quality monitor") you write "Air pollutants will be monitored at 2 classrooms and outdoor area of each school for 2 consecutive weeks (5 school days per week)". So, in the abstract you should say 10 days.

3. **Subjects and Study Methodology** (i) Is the proposed design and methodology appropriate for the study? (ii) Are sample sizes clear, justified, adequate and realistic? (iii) Are any preliminary data available? (iv) How feasible is the proposed timeframe?

> I am satisfied that (i), (ii), (iii), (iv) are addressed appropriately. Maybe you should add the number of participants in the abstract.

4. **Outcomes and Data Analysis** (i) Are the primary and secondary outcomes clearly defined? (ii) Have potential problems been anticipated and addressed? (iii) Is the statistical/analytical design appropriate and clearly explained?

> (i) The outcomes are clearly defined in the abstract, but not in the text. They are mentioned in section d) (iv) while you explain data analysis. I suggest that you include a specific section in the text.
> (ii) You will use NASA satellite data to estimate residential air pollution at a resolution of 1 km x 1 km, as you won't measure it directly in the homes. It seems that this large resolution will make it impossible to differentiate air pollution levels from one residential location or home to another. "Residential location" is not defined.
> (iii) The statistical analysis seems appropriate.

5. **Research Capability** Comment on (i) the research team's expertise and track record (incl. principal investigator / project team members / collaborators) and (ii) the existing facilities of the Institution where the research will be conducted.

> (i) Expert research team in this field.
> (ii) Existing facilities are mentioned in the application.

6. **Budget** Is the request for research personnel, consumables, equipment and overall budget justified and reasonable?

> Difficult to comment.

7. **Ethical and Safety Considerations** Is the proposed research ethically sound? Outline any safety or ethical issues that

from the proposed research and comment on whether these have been adequately addressed in the proposal. Has ethical approval been sought?

approval being sought. No issues.

8. **Overall Comments and Conclusion** It is always helpful for applicants to receive constructive feedback from reviewers. What are the specific strengths and weaknesses of this proposal? Please include a brief overall appraisal of the proposal focusing on any areas for improvement and the basis for your comments, e.g. awareness of other work in the field.

Strengths:

Last study about asthma prevalence one decade ago. Use of validated questionnaires. First study in Hong Kong trying to associate indoor air pollution in school and respiratory problems. Large sample size.

Weaknesses:

Results adjusted by an estimation of air pollution at residential locations.

REFEREE'S ASSESSMENT FORM

Reference No.: 12134021

Project Title: Prevalence and risk factors of childhood asthma, allergic rhinitis and eczema in – three surveys from 1995 to 2014

PART A: REFEREE'S DETAILED REMARKS ON THE INDIVIDUAL SECTIONS OF THE GRANT APPLICATION

This part, except Question 9, will be forwarded to the applicant for refining and improving the proposal. The identity of the reviewer will not be released to the applicant. Please provide as much detail as possible to assist the applicant and the Grant Review Board.
Please comment on the proposed research in the following aspects

1. **Originality and Impact** What is the importance of the proposed research in terms of its originality and potential impact in the area under study? Is the proposed work original and important and will it influence practice?

The study is not really original as it is repeating an previous study done in Hong Kong. However the updated study will allow an assessment of recent trends and whether changes in prevalence of asthma are related to changes in risk factors. Also the results have the potential to inform policies regarding the future locations of schools.

2. **Research Questions, Aims and Hypotheses** How specific, clearly expressed and realistic are the research questions, aims and hypotheses?

The research questions, aims and hypotheses are clearly stated and realistic. The revisions the authors have made have improved the proposal in this respect.

3. **Subjects and Study Methodology** (i) Is the proposed design and methodology appropriate for the study? (ii) Are sample sizes clear, justified, adequate and realistic? (iii) Are any preliminary data available? (iv) How feasible is the proposed timeframe?

The design and methodology are appropriate to accomplish the study aims. The sample size is justified and realistic. There are no preliminary data. The proposed timeframe seems realistic.

4. **Outcomes and Data Analysis** (i) Are the primary and secondary outcomes clearly defined? (ii) Have potential problems been anticipated and addressed? (iii) Is the statistical/analytical design appropriate and clearly explained?

The outcomes are clearly defined. The authors propose to use 'maternal education attained' as the sole indicator of socio-economic status. This requires some justification, especially since this variable will apparently have only four categories. The authors intend to adjust for the lack of independence between students using a 'marginal model' which is appropriate. However they state that district will be used as the stratum when in this case the individual school should be used as the stratum as multiple schools per district are to be chosen and school level factors are most likely to induce correlation in outcomes between students.

5. **Research Capability** Comment on (i) the research team's expertise and track record (incl. principal investigator / project team members / collaborators) and (ii) the existing facilities of the Institution where the research will be conducted.

The research team appears to have the expertise required to successfully carry out the study.

6. **Budget** Is the request for research personnel, consumables, equipment and overall budget justified and reasonable?

Yes seems reasonable. Previous queries have been addressed in the revision.

7. **Ethical and Safety Considerations** Is the proposed research ethically sound? Outline any safety or ethical issues that from the proposed research and comment on whether these have been adequately addressed in the proposal. Has ethical approval been sought?

Yes the research is ethically sound.

8. **Overall Comments and Conclusion** It is always helpful for applicants to receive constructive feedback from reviewers. What are the specific strengths and weaknesses of this proposal? Please include a brief overall appraisal of the proposal focusing on any areas for improvement and the basis for your comments, e.g. awareness of other work in the field.

Strengths:

The project addresses an important disease which accounts for a good deal of childhood morbidity in Hong Kong and whose prevalence has been increasing.<br>The study will allow assessment of recent trends in asthma prevalence and possible risk factors accounting for likely increases in prevalence.<br>An experience research team and a validated questionnaire.

Weaknesses:

Health outcomes are assessed by parents and may not always be accurate.
Some aspects of statistical analysis still need to be clarified.

REFEREE'S ASSESSMENT FORM

Reference No.: 12134021

Project Title: Prevalence and risk factors of childhood asthma, allergic rhinitis and eczema in – three surveys from 1995 to 2014

PART A: REFEREE'S DETAILED REMARKS ON THE INDIVIDUAL SECTIONS OF THE GRANT APPLICATION

This part, except Question 9, will be forwarded to the applicant for refining and improving the proposal. The identity of the reviewer will not be released to the applicant. Please provide as much detail as possible to assist the applicant and the Grant Review Board.
Please comment on the proposed research in the following aspects

1. **Originality and Impact** What is the importance of the proposed research in terms of its originality and potential impact in the area under study? Is the proposed work original and important and will it influence practice?

> The topic is definitely important. Data on prevalence of these common conditions are necessary for planning of services for children suffering from these diseases. There have been many similar prevalence studies done around the world using similar methodologies. The proposed tools are useful in estimating prevalence in the community but these tools are not accurate enough to confirm diagnoses on an individual basis. It is well-known and well documented in Hong Kong and around the world that increasing air pollution can precipitate asthma exacerbations. By nature of the cross-sectional design and the rather imprecise tools for making accurate diagnosis, the results of the study will unlikely have any major impact on the prevention or treatment of asthma and related allergic conditions.

2. **Research Questions, Aims and Hypotheses** How specific, clearly expressed and realistic are the research questions, aims and hypotheses?

> Two hypotheses were clearly stated. Hypothesis 1 can be tested using the proposed methodology. However, given the fact that most similar studies in the developed world from 1995 to 2008 have not shown any significant increase in the prevalence of the three diseases in question, one would be very surprised if there might be any increase in the studied population since 2001. The second hypothesis is problematic. The cross sectional nature of the study would not be the appropriate method to confirm the relationship of diseases and the exposure in the individuals. Personal exposure to pollutants are always difficult to obtain and one such gold standard is to use personal sampler as it will reflect the personal exposure much better than data obtained from other sources.
>
> Hypothesis 2 as stated is rather imprecise that we are not sure what air pollutants that the investigators are interested at. Different pollutants are drastically different depending on the location of exposure. Some are more related to indoor exposure while others are more in the outdoor environment. The "dose" of exposure also depends on the level of physical activity and the duration spent outdoor. For example, studies in the US have demonstrated the detrimental effects of outdoor air pollutants such as ozone may only be more important in those children exercise outdoor in polluted areas. The proposed method in studying 4000 children (> 90% of them have no asthma) is very unlikely to generate any new information to test this second hypothesis.

3. **Subjects and Study Methodology** (i) Is the proposed design and methodology appropriate for the study? (ii) Are sample sizes clear, justified, adequate and realistic? (iii) Are any preliminary data available? (iv) How feasible is the proposed timeframe?

> The sample size is reasonable for estimating the prevalence of the community but the investigators should be aware of the fact that the methodology used is not accurate in documenting diseases in individuals. In particular, validation studies have demonstrated very poor sensitivity and specificity especially for allergic rhinitis and eczema. The proposed time frame is appropriate for the proposed work.

4. **Outcomes and Data Analysis** (i) Are the primary and secondary outcomes clearly defined? (ii) Have potential problems been anticipated and addressed? (iii) Is the statistical/analytical design appropriate and clearly explained?

> There is no major problem in estimating the prevalence rates (hypothesis 1) as long as the investigators are aware of the limitations. With such sample size, the current design, the complexity of estimation of exposure to various air pollutants, I do not see how it may generate any new or exciting information for the understanding of the problem of air pollution and allergic diseases.
>
> For example, is exposure in early life more important than current exposure? Do we have information on past exposure?

5. **Research Capability** Comment on (i) the research team's expertise and track record (incl. principal investigator / project team members / collaborators) and (ii) the existing facilities of the Institution where the research will be conducted.

> The team has experienced in conducting similar epidemiology studies in estimating prevalence of asthma/allergic diseases but they may underestimate the complexity of studying relationship of air pollution and airway diseases.

6. **Budget** Is the request for research personnel, consumables, equipment and overall budget justified and reasonable?

Appropriate.

7. **Ethical and Safety Considerations** Is the proposed research ethically sound? Outline any safety or ethical issues that from the proposed research and comment on whether these have been adequately addressed in the proposal. Has ethical approval been sought?

None

8. **Overall Comments and Conclusion** It is always helpful for applicants to receive constructive feedback from reviewers. What are the specific strengths and weaknesses of this proposal? Please include a brief overall appraisal of the proposal focusing on any areas for improvement and the basis for your comments, e.g. awareness of other work in the field.

Strengths:

The team has experience in conducting such prevalence study using the proposed methodologies.

Weaknesses:

Given what we know about exposure to air pollution and allergic diseases, studying the occurrence of diseases measured by imprecise tools and correlating with the one time (2014) estimation of the various air pollutants will very likely lead to any meaningful conclusion.

Project No.: 12134021
Project Title : Prevalence and risk factors of childhood asthma, allergic rhinitis and eczema in – three surveys from 1995 to 2014.

GRB

GRB1: Pilot the new questions before implementing them in the study.

A: *We will conduct a pilot test to test out the new questions at the paediatric outpatient department of Queen Mary Hospital. Parents of children attending the clinic will be invited to participate.*

GRB2: It is appropriate to use multi-level modelling but the team should use "school" rather than "district" as stratum. Multiple schools per district would be chosen and school level factors are most likely to induce correlation in outcomes between students.

A: *We agree that multiple schools per district should be chosen and individual school level instead of district should be used as the stratum for the analysis model. The statistical method section had been revised.*

GRB3: Consider adjusting the analysis by the socioeconomic status of the family and not just maternal education.

A: *We concur that only maternal education for the socio-economic status of the family is not sufficient for analysis, therefore, questions for family income, family size, area of living quarter and the type of accommodation will be added in the questionnaire. All these variables will be used for the analysis. The data collection section, analysis method and the English and Chinese questionnaire had been revised.*

GRB4: Consider the risk of Type I errors as there would be a large number of outcomes to be assessed.

A: *Bonferroni adjustment will be used to reduce the risk of Type I errors and tackle the multi-testing issue. The analysis method had been revised.*

GRB5: Provide evidence of regulatory approval (including survey research ethics).

A: *Our study has already received approval from the Institutional Review Board of the University of Hong Kong/Hospital Authority Hong Kong West Cluster. An approval letter will submitted to the research council.*

GRB6: An audit fee of $5,000 should be included under "Other Expenses".

A: *We will add $5,000 audit fee into the budget and the budget had been revised.*

GRB7: A governance system to adequately monitor the disbursement of cash or cash vouchers to ensure accountability and traceability is required.

A: *Dr SL lee is an honorary Clinical Associate Professor of the Department of Paediatrics and Adolescent Medicine. Professor YL Lau and Mr Wilfred Wong are university staff. Thus, the funding received will enter the Research Account under the Department of Paediatrics and Adolescent Medicine, the University of Hong Kong. The disbursement of cash will follow the HKU regulation. The usual governance system will monitor the budget usage, with the head of the Department countersigning the disbursement of cash. An approval letter from the head of the department for supporting our research study and willing to control the cash flow is attached for reference.*

GRB8: Budget items Ozone monitor (ZDL-1200) ($59,520) and Sulphur Dioxide monitor (ZDL-1300) ($49,560) should be included under "Equipment" instead of "Other Expenses". Please submit revised budget as per the template under the point-by-point response..

A: *Budget for Ozone monitor and Sulphur Dioxide monitor had been relocated to the item under "Equipment". A revised budget will be submitted to the research office.*

GRB9: The group led by Dr. Lee has several proposals recommended for support in the current open call: 0213316 – [Functional analysis and evaluation of ultrastructure of respiratory cilia in healthy Chinese children in Hong Kong]; 12134021 – [Prevalence and risk factors of childhood asthma, allergic rhinitis and eczema in – three surveys from 1995 to 2014]. Provide assurance that each project can be completed according to the proposed timeline and resources. Justify the expensive for these projects on consumables and manpower in more detail and clarify if any savings can be obtained from conducting these studies in parallel if they are subsequently approved.

A: *Our department will have two different research teams of staff to support Dr Lee's studies to ensure the complete date of the study is within the proposed timeline. The co-investigators of Dr Lee in both projects are also very experienced and collaborated well in the past. They will help to supervise the project. For the current project, we have submitted the timetable of work as followed. For first 3 months, a full time research assistant will help to print the letters to school and parents, print and label questionnaires, prepare return envelopes, pack small gifts for participants and conduct pilot test for the newly added questions. She/he will also help Mr. Wilfred Wong to order equipment and learn to operate the equipment and download the data from the equipment. For the next 3 months, she/he will help to generate a random school list and contact the selected school for agreement to participate in the study. She/he will liaise with schools for the period of visit. For the next 12 months, she/he will visit the schools, distribute the questionnaires, set up equipment at school and pay regular visits to the school to download air pollutants data and collect the questionnaires. For the last 6 months, she/he will help with data entry, handling of data, data analysis and report writing under the supervision of Mr Wilfred Wong, Dr SL Lee and Professor Lau. We need another research assistant for months to assist Dr HK Lai to analyze satellite data and correlate with questionnaire and data from air pollutants monitors. The main skill set that the research assistants require in this project is on field study, operation of air quality monitor and handling large dataset. This will be different from the laboratory-based skill for the project 0213316 – [Functional analysis and evaluation of ultrastructure of respiratory cilia in healthy Chinese children in Hong Kong] In addition, the equipment and consumables required are different for the two projects. Thus, savings from conducting these studies in parallel will be very difficult, if not infeasible.*
